# Supplementary material for: 2D honeycomb transformation into dodecagonal quasicrystals driven by electrostatic forces
Source: Nat Commun. 2022 Dec 7;13:7542. doi: 10.1038/s41467-022-35308-z (PMC9729568; doi:10.1038/s41467-022-35308-z)
Supplement: Supplementary file 1 — Supplementary Information [file 41467_2022_35308_MOESM1_ESM.pdf]

Supplementary information

2D honeycomb transformation into dodecagonal quasicrystals driven by electrostatic forces

S. Schenk et al.

Supplementary Figures

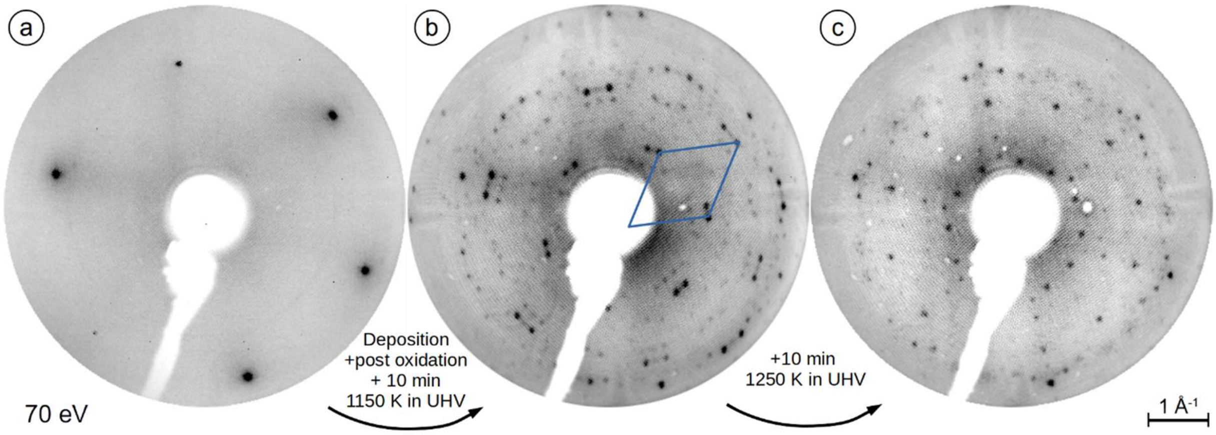

Supplementary Figure 1 | **Structural transition in ultrathin Sr-Ti-O films on Pt(111) upon high temperature reduction.** Series of low-energy electron diffraction (LEED) pattern illustrating the structural evolution in ultrathin Sr-Ti-O on Pt(111). (a) The bare Pt(111) substrate. (b) Precursor structures observed upon annealing the deposited and post-oxidized material for 10 min at 1150 K. The blue rhombus emphasizes the unit cell of a rotated honeycomb structure. (c) The 48:18:6 approximant of Sr-Ti-O on Pt(111) formed from the precursor upon additional 10 min UHV annealing at 1250 K.

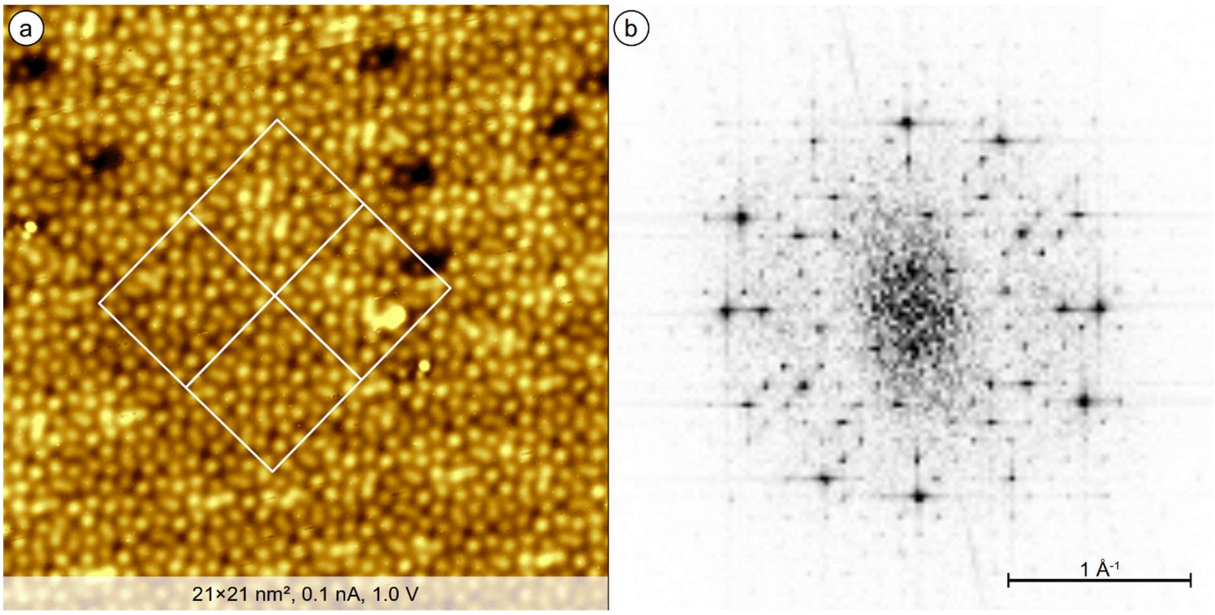

Supplementary Figure 2 | **Local atomic structure of 48:18:6 approximant seen by STM.** (a) Larger scale STM data of the 48:18:6 approximant. Single domain of the approximant structure in atomic resolution. Horizontal and vertical lines indicate the presence of periodic long-range ordering. Four copies of the unit cell are marked in white. (b) Fourier transform (FT) of the STM image shown in a. All spots of the FT reside on a small square grid. The twelve most intense spots at an in-plane lattice vector magnitude of about 1 $Å^{-1}$ from the origin closely resemble the pattern of the dodecagonal oxide quasicrystal. However, by residing on the square lattice they differ in their distance to the origin and are not separated by exactly 30°, which is the case for the truly dodecagonal pattern.

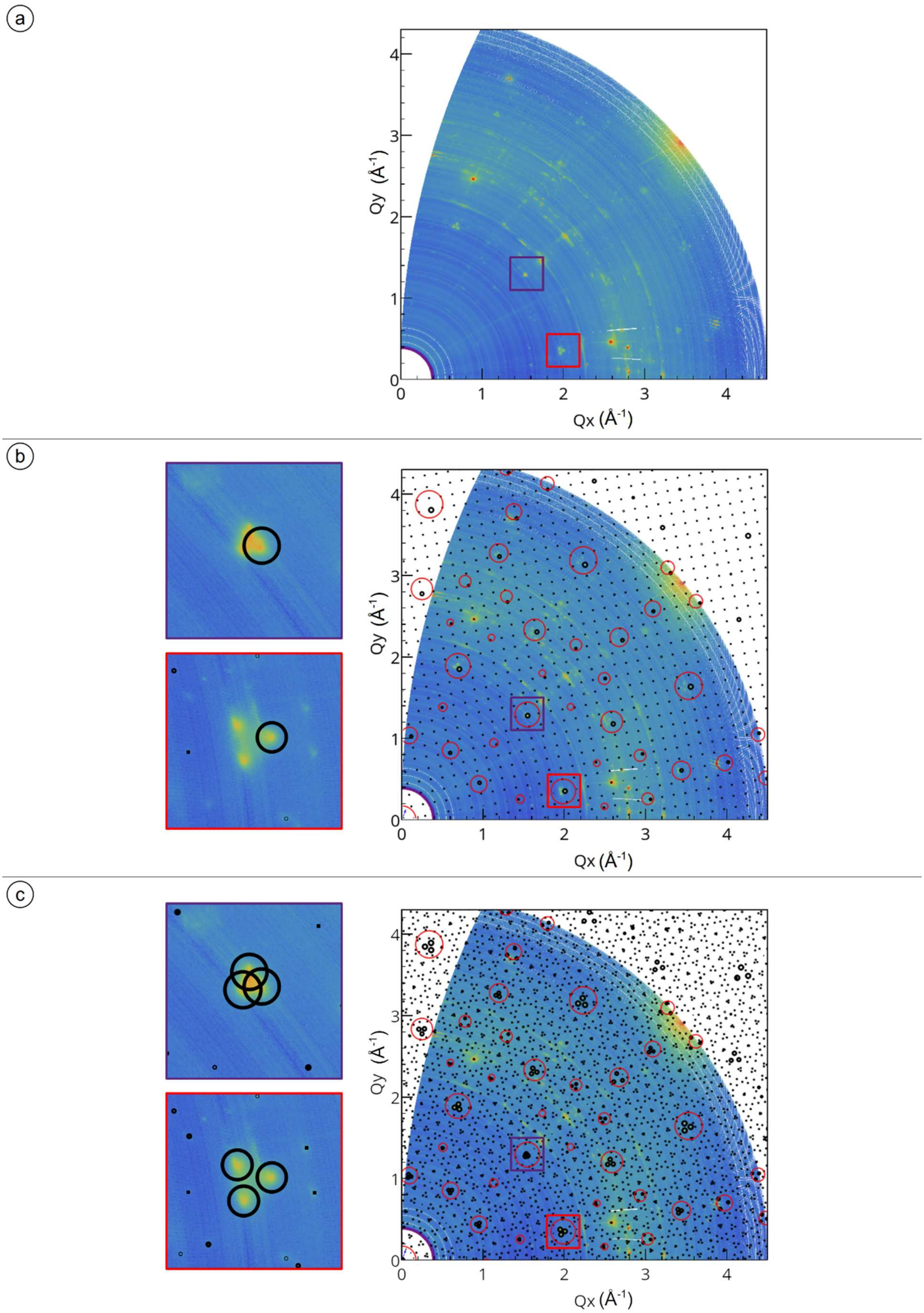

Supplementary Figure 3 | **Reciprocal space map of the 48:18:6 approximant in Sr-Ti-O on Pt(111).** a) Six domains of the two-dimensional ternary oxide layer contribute to the diffraction pattern resulting in triangular splitting of neighbouring spots. b) Black circles mark the positions of all peaks of a single domain of the commensurate superstructure. The relative spot intensities are encoded in the circle diameter. c) Full indexing of the diffraction pattern using three rotational and three mirror domains of the almost rectangular structure. These domains are induced by the substrate symmetry.

Supplementary Notes

SXRD measurements

SXRD experiments were performed using an UHV diffractometer operated in the Z-axis mode [S1]. Integrated reflection intensities were collected under grazing incidence of the incoming beam (λ=1.13 Å) close to the critical angle of total reflection for the Pt(111) surface. Supplementary Figure 4 shows a representative 2D pixel detector image for the (7 12 L) reflection of the Sr-Ti-O 48:18:6 approximant on Pt(111).

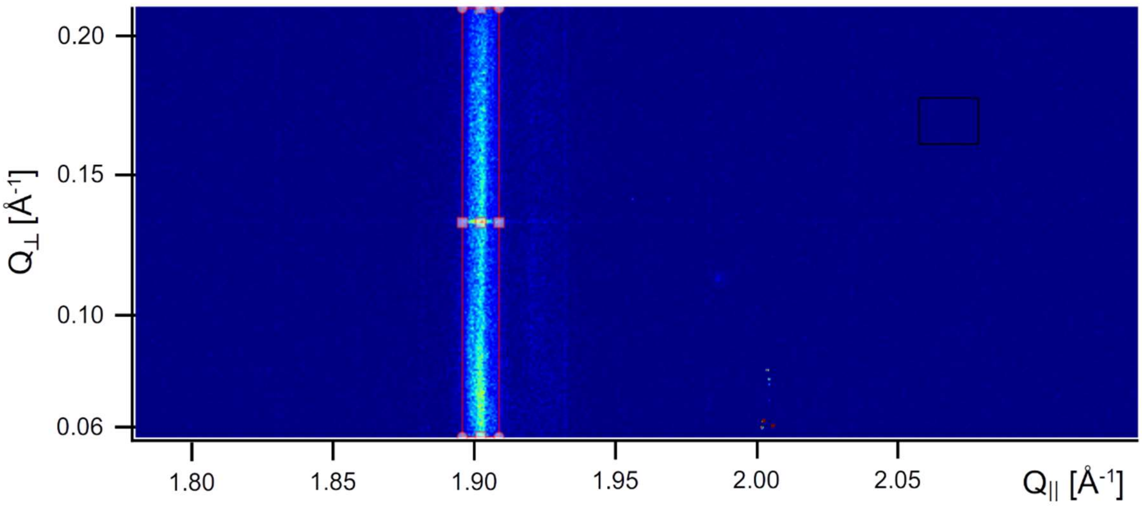

Supplementary Figure 4 | **SXRD raw data for ultrathin Sr-Ti-O on Pt(111).** Representative 2D detector image having a width of 560 x 240 pixels corresponding to a total parallel and perpendicular momentum of 0.354 and 0.155 Å-1, respectively.

The full width at half maximum (Γ) of the reflection along the parallel momentum transfer (Q_{par}) is equal to 0.0046 ± 0.0001 $Å^{-1}$ which provides an estimated domain size of the order of 2π/Γ, which is equal to ~1350 Å indicating the long-range two-dimensional ordering of the film.

Within the recorded SXRD reciprocal space maps the bulk truncation rods and the surface-related signals are disturbed by x-ray scattering from unavoidable defects in the bulk of the Pt substrate. Thus, we developed a custom routine for filtering undisturbed surface diffraction spots and for proper background subtraction. In this routine, the background signal of a given diffraction spot is analysed based on four background areas as depicted in Supplementary Figure 5. In a first step, the four background areas are compared to each other and areas of unexpected high intensities (area intensity larger than twice the minimal intensity of the four areas) are ignored.

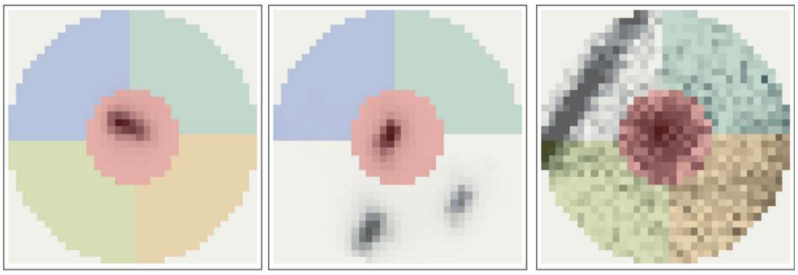

Supplementary Figure 5| **Analysis of background regions around SXRD spot intensities.** Regions around three different diffraction spots (left to right) are divided in a central segment (red) and four surrounding background areas (other colors). Uncoloured background areas have been excluded for background evaluation.

In a second step, background areas where the intensity per pixel varies strongly (their standard deviation differs by more than 30% compared to the average standard deviation of all background areas around a given diffraction spot) are ignored. Examples for this filtering are given in Supplementary Figure 5. The coloured segments indicate the regions that have been considered for background subtraction. The peak area has been determined from integrating the intensity within the central red region followed by subtracting the mean intensity calculated from the valid background areas.

From 2750 analysed spots positions in the recorded reciprocal space map, 460 spots intensities have been accepted for further analysis reducing to 182 symmetry-independent intensities by averaging over symmetry equivalent ones. The average agreement factor over symmetry equivalent reflections is in the 10% range which provides an estimate for the systematic uncertainty. The total 1σ uncertainty is then derived by the quadrature sum of the statistical and the systematic uncertainty [S2]. The structure refinement was carried out after applying instrumental correction factors to the reflection intensities [S3] to obtain the experimental structure factor magnitudes, |Fobs|, by using the Software package "SHELX" [S4] for performing a weighted fit in which the goodness of fit (GOF) and the weighted residuum (wR2) are used as agreement factors [S4, S5]. The best fit model, which is reported in the main text, has been obtained from an initial configuration in which Sr atoms occupy the vertex positions obtained from STM measurements. The positions of the Ti atoms can then be derived by calculating the difference Fourier synthesis. These are close to those of the proposed model. In the final refinement, the positions of the Sr and Ti atoms were allowed to vary using constraints on the Sr-Ti and Ti-Ti distances which were allowed to vary from their mean distance (3.19 and 3.65 Å) by a standard deviation of 0.25 Å. For the best fit model we obtain wR2=0.21, GOF=1.50 and R1=0.11, the latter being the average of the relative deviation between observed and calculated structure factor

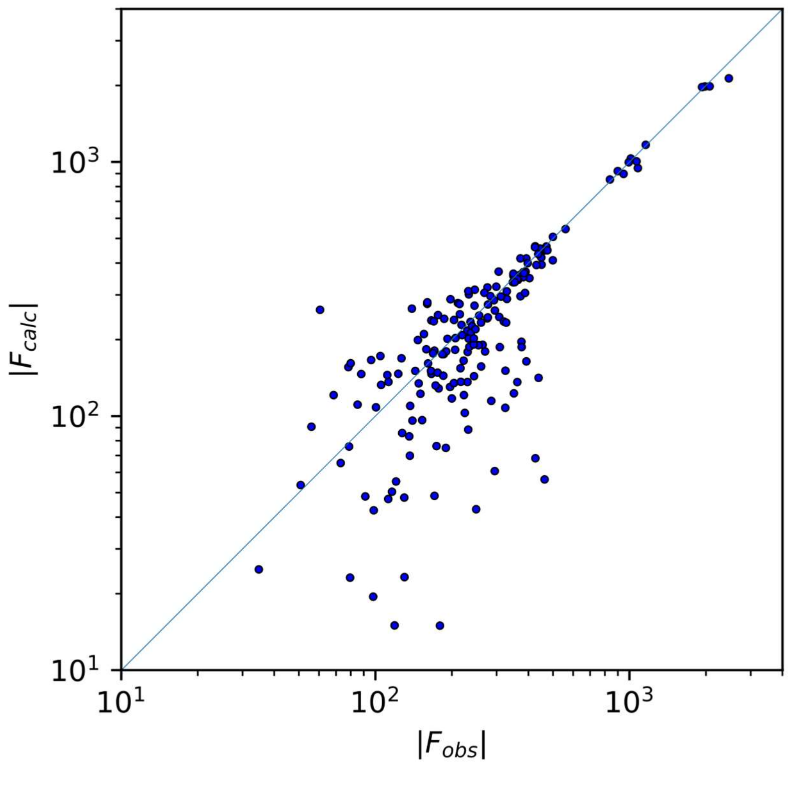

Supplementary Figure 6 | **Comparison of the measured structure factor amplitude with the best fit model.** 179 symmetry-independent intensities from the SHELXL optimization are shown as blue dots. The light blue line indicates, where $|F_{obs}|$ equals $|F_{calc}|$. 3 spots with lower $|F_{calc}|$ have been omitted in the plot.

magnitudes. Supplementary Figure 6 compares the observed with the calculated structure factor magnitudes.

Supplementary Figure 7a shows the relaxed positions of the Sr atoms as green solid circles, whereas empty circles mark the high-symmetry positions assuming unperturbed prototiles, which have been taken as starting positions. In general, we find deviations of the Ti positions along both, x and y, which involves slight deviations from the perfect tiling geometry. Consequently, the Ti positions within different Sr squares and different Sr triangles were allowed to vary. One example for the optimization scheme of the Ti atom positions is provided on the basis of Supplementary Figure 7. The Ti atom positions have been varied as indicated in Supp. Fig. 7a by the red arrows for the square and by black arrows for the triangle. In the square, the position of the four Ti atoms has been varied symmetrically between the edge and the center of the square (four red arrows). Analogously, the Ti atom position within the triangle is allowed to vary between the center and the edge of the triangle (two black arrows). SHELXL was used to calculate the unweighted residuum R1 for these variations as depicted in Supplementary Figure 7b as contour plot. Along the Y axis (red) the variations from the square edge inwards are plotted, while the movement of the Ti atoms in the triangles is plotted on the x axis. This R-factor map exhibits a clear minimum (white star in Supplementary Figure 7b), where the corresponding R1 decreases to 0.26. These optimized Ti positions for a symmetric decoration are shown in (a). They result in six different Ti-Ti distances depending on the environment of the given tiling element. With TX denoting the number of triangles X surrounding a given triangle in the tiling we find: 3.21 Å between Ti atoms in a square and a triangle T0 (or T2), 2.92 Å between two neighbouring Ti atoms in the square, 3.42 Å between Ti atoms in a square and a triangle T1, 3.05 Å between Ti atoms in two triangles T1, 3.06 Å for Ti atoms in a rhombus and a triangle T1 and 3.43 Å between Ti atoms in triangles T1 and T2.

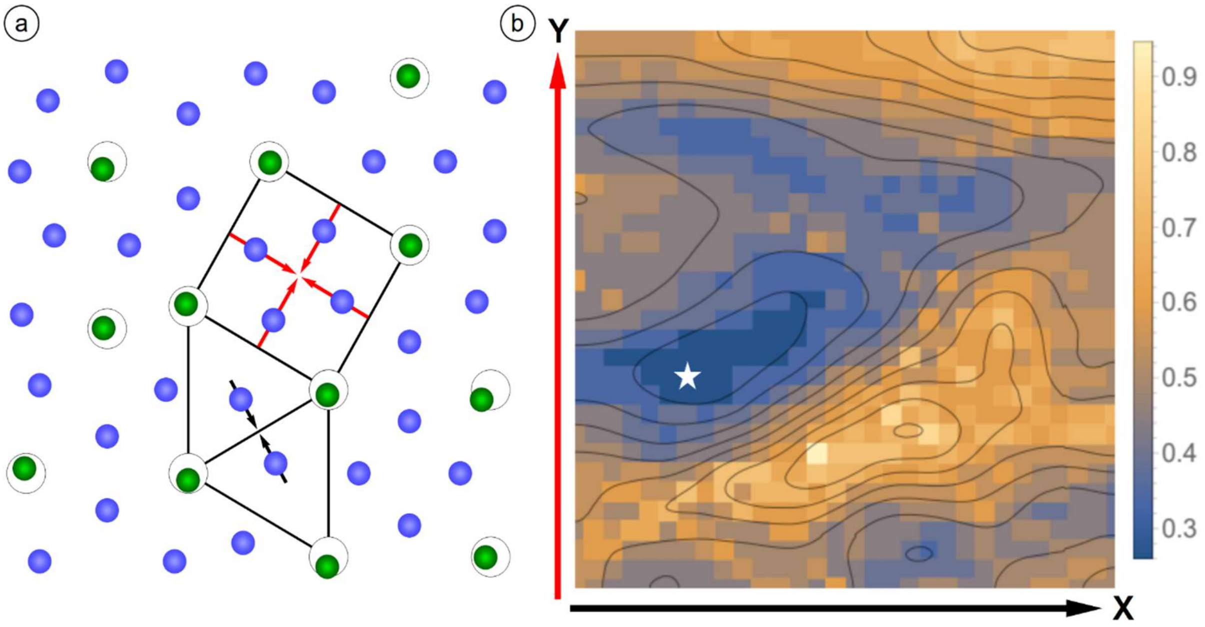

Supplementary Figure 7 | **Systematic variation of Ti atomic positions within the square and the triangle tiling elements.** (a) The Ti atoms of the asymmetric unit marked in blue. Their position was varied along the black arrow in each triangle, which corresponds to the x-axis in (b). Variation along the red arrow within the square tiles corresponds to the y-axis in (b). Previously refined Sr atoms marked in green. Vertices of the tiling elements are shown as black open circles. (b) Contour plot of R1 versus x and y, the positions of Ti atoms along the arrows within the triangles and squares, respectively. The white star indicates the minimum position that has been adopted for the positions of the Ti atoms in (a) 0.4 Å from the center of the triangle and 1.3 Å from the square edge. The length of the arrows of (a) span the full axis in (b), which corresponds to 3.4 (red) and 2.0 Å (black).

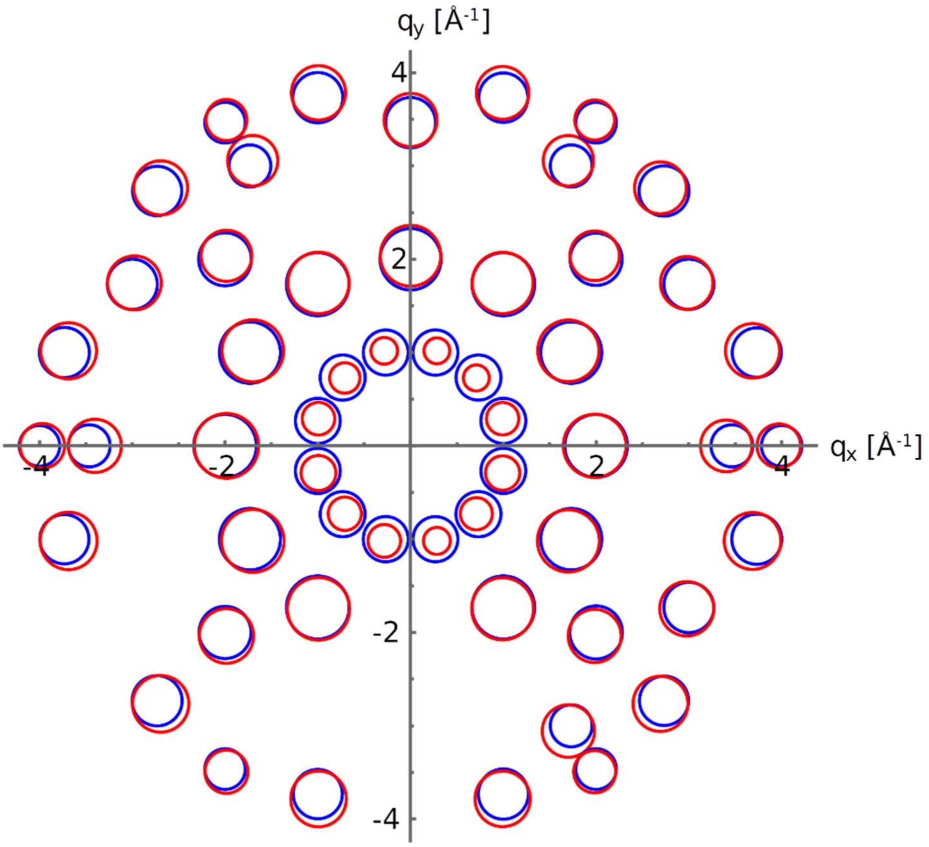

Supplementary Figure 8 | **Comparison of SXRD intensities of the 48:18:6 approximant and the OQC.** The logarithm of the squared structure factor magnitude of the OQC is given as the area of the blue circles. For comparison the squared structure factor amplitude of the approximant diffraction spots within a maximum distance to the OQC spot positions of 0.1 Å^{-1} are superimposed in red.

Supplementary Figure 8 compares the diffraction intensities of the 48:18:6 approximant in Sr-Ti-O/Pt(111) with those measured for the OQC in Ba-Ti-O/Pt(111). In this reciprocal space map only experimentally determined diffraction orders of non-zero intensity for the OQC, given in blue circles, for which comparable data for the approximant (red circles) are available, are shown. Considering the different A-type atoms in both systems, the good match of the squared structure factor magnitudes between the two structures is astonishing. Due to the existence of a periodic lattice for the approximant, the spot positions vary by different amounts from those of the OQC, which must be accompanied by an intensity decrease with increasing distance. The close resemblance of both patterns underlines the general applicability of the tiling decoration scheme proposed in the main paper in Fig. 3.

DFT calculations

For the honeycomb (Ba)-Ti-O calculations, we used supercells with a ≈ 20.4 Å and for the sigma phase calculations we used supercells with a ≈ 19.0 Å, designed to match the compositions. For the calculations comparing these two Ba-Ti-O approximants, we used a trilayer of Pt. The bottom layer was kept fixed, the second layer was allowed to relax only along the z direction, and the top layer was allowed to relax fully. The experimental Sr-Ti-O 48:18:6 approximant is so large that only one fixed layer of Pt was included. The total periodic distance along z was 20 Å for all structures, ensuring a vacuum layer of at least 10 Å thickness.

A Γ-centered 1x1x1 k-point grid was used for the large Sr-Ti-O approximant and a Γ–centered 3x3x1 grid for the smaller approximants. Following the recommendation of Hu and Metiu [S6], we set U = 2 eV for Ti. Test calculations with dipole corrections showed little difference between the different structures considered at a cost of poor convergence, so dipole corrections were not incorporated. For dispersion, we used the nonlocal density functional optB86b-vdW [S7].

Optimizing the strain of a thin film oxide is challenging. Our procedure was as follows. We only considered isotropic in-plane strains for the hexagonal (square) planar cells. We selected an initial guess for the stain. For this strain, there is an ideal number of Pt atoms per layer per cell based on the Pt lattice parameter. We determined the strained commensurate Pt geometries that most closely matched the chosen lattice having integer number of Pt per layer greater than and less than the ideal value. The energies of the relaxed Pt-Ba-Ti-O systems and the energies of the Pt trilayer alone (reoptimized) were calculated, and the (Ba-Ti-O plus binding) energies were calculated as the difference between the two as was done in [S8]. The (Ba-Ti-O plus binding) energy at the experimental Pt lattice parameter was estimated via linear interpolation with respect to Pt count per layer per cell. To optimize the strain unambiguously, we used DFT-calculated stress rather than binding energies. From calculations done at different strains, we determined the Ba-Ti-O strain where the average in-plane Pt-Ba-Ti-O internal stress at the experimental Pt lattice constant was equal to the interpolated in-plane stress of Pt alone.

The DFT relaxation used to determine the atomic positions shown in Figure 4 of the main paper began with a symmetric $Ba_4Ti_{100}O_{151}$ cell with $a \approx 50$ Å, $b \approx 29$ Å and c= 20 Å. To save time and avoid symmetry breaking, no Pt substrate was included. Instead, the Ba, Ti, and O heights were fixed at the average values found for the sigma phase. The cell parameter *c* was fixed at 20 Å, but the *a* and *b* cell parameters were allowed to relax. The number of valence electrons (NELECT in VASP) was set corresponding to ionic charges of +2, +3, and -2 for Ba, Ti, and O, respectively. VASP adds a uniform background charge to ensure overall charge neutrality.

Since alkaline-earth metals are known to form stable peroxide species [S9], we considered a modification of the Cockayne model [S10] where nearby pairs of oxygen atoms in the n=10 rings were replaced by peroxide species at the centers of the rings. DFT calculations show that in the absence of Sr this structure is indeed (meta)stable. However, when Sr is added to the model, the peroxides species dissociate and the structure relaxes to the original model.

Supplementary References

[S1] Brennan, S. & Eisenberger, P., A novel X-ray scattering diffractometer for studying surface structures under UHV conditions. Nucl. lnstrum. Methods A**222**, 164-167 (1984).

[S2] Robinson, I.K. & Tweet, D.J., Surface X-Ray Diffraction. Reports on Progress in Physics **55**, 599 (1992).

[S3] Drnec, J., Zhou, T., Pintea, S., Onderwaater, w., Vlieg, E., Renaud G., and Felici, R., Integration techniques for surface X-ray diffraction data obtained with a two-dimensional detector. J. Appl. Cryst. **47**, 365–377 (1994).

[S4] Sheldrick, G. M., Crystal structure refinement with SHELXL. Acta Cryst. C**71**, 3-8 (2015).

[S5] Abrahams, A. C., Indicators of Accuracy in Structure Factor Measurement. Acta Cryst. A**25**, 165 (1969).

[S6] Liechtenstein, A. I., Anisimov, V. I. & Zaanen, J. Density-functional theory and strong interactions: Orbital ordering in Mott-Hubbard insulators. Physical Review B **52**, R5467–R5470 (1995).

[S7] Dion, M., Rydberg, H., Schröder, E., Langreth, D. C. & Lundqvist, B. I. Van der Waals Density Functional for General Geometries. Physical Review Letters **92** (2004).

[S8] Wang, S., Hu, X. Goniakowski, J., Noguera, C., and Castell, M. R., Influence of the support on stabilizing local defects in strained monolayer oxide films. Nanoscale **11**, 2412–2422 (2019).

[S9] Wiberg, E., Wiberg, N., and Holleman, A. N. Inorganic Chemistry, Academic Press 2001, ISBN 0-12-352651-5, pp. 471–502.

[S10] Cockayne, E., Mihalkovič, M., and Henley, C. L., Structure of periodic crystals and quasicrystals in ultrathin films of Ba-Ti-O. Phys. Rev. B **93**, 020101(R) (2016).
